# The link between broiler flock heterogeneity and cecal microbiome composition

**DOI:** 10.1186/s42523-021-00110-7

**Published:** 2021-07-31

**Authors:** Randi Lundberg, Christian Scharch, Dorthe Sandvang

**Affiliations:** 1 Chr. Hansen A/S, Boege Allé 10-12, 2970 Hoersholm, Denmark; 2 Feedtest, Löbejüner Straße 32, 06193 Wettin-Löbejün, Germany

**Keywords:** Broiler production, Broiler performance, Cecal microbiome, Flock uniformity, Flock heterogeneity, Metagenomics, Poultry microbiome

## Abstract

**Background:**

Despite low genetic variation of broilers and deployment of considerate management practices, there still exists considerable body weight (BW) heterogeneity within broiler flocks which adversely affects the commercial value. The purpose of this study was to investigate the role of the cecal microbiome in weight differences between animals. Understanding how the gut microbiome may contribute to flock heterogeneity helps to pave the road for identifying methods to improve flock uniformity and performance.

**Results:**

Two hundred eighteen male broiler chicks were housed in the same pen, reared for 37 days, and at study end the 25 birds with highest BW (Big) and the 25 birds with lowest BW (Small) were selected for microbiome analysis. Cecal contents were analyzed by a hybrid metagenomic sequencing approach combining long and short read sequencing. We found that Big birds displayed higher microbial alpha diversity, higher microbiome uniformity (i.e. lower beta diversity within the group of Big birds), higher levels of SCFA-producing and health-associated bacterial taxa such as *Lachnospiraceae*, *Faecalibacterium*, *Butyricicoccus* and *Christensenellales,* and lower levels of *Akkermansia muciniphila* and *Escherichia coli* as compared to Small birds.

**Conclusion:**

Cecal microbiome characteristics could be linked to the size of broiler chickens. Differences in alpha diversity, beta diversity and taxa abundances all seem to be directly associated with growth differences observed in an otherwise similar broiler flock.

**Supplementary Information:**

The online version contains supplementary material available at 10.1186/s42523-021-00110-7.

## Background

Broiler production is based upon a multiple-generation procedure of purebred genetic lines and their crosses. Broiler purebred lines have low heterozygosity and are very closely related to each other [1]. Intensive selection processes over the past five decades have decreased genetic variation within purebred lines resulting in a distinctly low genetic variation of the broiler. When housed under the same conditions and fed the same feed, one should think that broilers would have a relatively comparable growth and that a certain uniformity in final body weight (BW) could be achieved. Nevertheless, at slaughter, variations in BW of 11–18% (Coefficient of variation (CV) of BW) in mixed sex flocks are regularly observed [2], and 8–10% have been reported even for male-only flocks [3]. Poor uniformity translates to decreased profitability due to devaluation of carcasses not complying with the processing plant and market specifications. At the same time, it is desirable to achieve healthy, productive birds reaching a high final BW. The low genetic variation is not expected to solely drive this variation in final BW, hence other factors must also play a role. Poor management practices or health problems can cause some birds to have reduced access to feed and water, but the problem with suboptimal carcass uniformity is not fully understood [2]. The gut microbiome, referring to the collective assembly of microbial organisms inhabiting the gastrointestinal tract and the functional potential of their genomes, is a player to also consider with regards to carcass uniformity. The composition and activity of the gut microbiome is predominantly shaped by dietary and environmental factors and to a smaller extent by host genetics [4, 5] and is known to impact animal health and productivity [6, 7]. Many studies have investigated the link between chicken productivity and the gut microbiota as was recently reviewed by Carrasco et al. [8]. The *Lachnospiraceae* has for instance, consistently been associated with high chicken productivity [8], possibly due to the anti-inflammatory potential of this short chain fatty acid (SCFA) producing family [9]. Lactic acid bacteria are also associated with chicken performance [8]. On the other hand, do the genus *Escherichia* and the family *Enterobacteriaceae* correlate frequently with low productivity due to a high pathogenic potential within these taxa [8]. *Enterobacteriaceae* is recognized as a pro-inflammatory marker of imbalance of the gut microbiota (dysbiosis) in poultry [10]. A few studies have investigated the cecal microbiome of birds of extreme BWs. Lee et al. investigated the cecal microbiota of 12 male and 12 female broiler chickens by 16S rRNA sequencing and found the genera *Faecalibacterium* and *Shuttleworthia* to be enriched in male chickens with the highest BW after 35 days of rearing [11]. Han et al. found that *Streptococcus* and *Akkermansia* correlated negatively with BW in cecum, whereas *Bifidobacterium* and *Lactococcus* in ileum and cecum respectively showed a positive correlation [12].

Designing appropriate microbiome trials can be challenging. A plethora of factors are involved in shaping the microbial gut community. If not carefully considered and controlled, these influences can confound the study [13, 14]. Animals housed together share microbiomes to a large extent due to the shared local environment and behaviours such as coprophagy and pecking/preening activities. This can result in a so-called cage or pen effect [14, 15], meaning that a pen-specific microbiome is developed within a single pen of animals. This phenomenon is a potentially confounding factor that may mask the effect under study, e.g. the effect of a feed additive intervention on the microbiome [14]. A sound solution is to spread out the confounding variable across a statistically appropriate number of pens replicated in the trial design and either housing the animals individually, pooling samples from each pen or subsampling one or more birds from each pen. However, the downside of these approaches are that they do not reflect the real-life situation of broiler chickens. Broilers are managed in flocks of several thousand within a single barn, sharing the same environment and consuming the same homogeneous feed. The considerable bird-to-bird variation existing within these flocks can obviously not be explained by pen-to-pen variation. In any group of broilers with *n* > 1, differences in BW are likely to be observed. A Gaussian distribution of weights is furthermore expected in big groups of birds (hundreds and more). We asked whether any differences in BW in a homogeneously reared group would coincide with variations in the microbiome; and if so, which differences. Therefore we designed a study to address the relation between varying final BW of broilers and their cecal microbiome composition when housed as a single flock in a barn. To this end, 218 male newly hatched Ross 308 broiler chicks were placed in one pen and reared for 37 days. The group size was chosen to be big enough to mimic the commercial production management situation, though still on a smaller, more manageable and controlled scale. Based on BW, the 25 heaviest (designated Big) and 25 lightest (designated Small) birds were selected, sacrificed and contents from the cecal sacs were sampled. Investigation of the ceca were chosen due to their high microbial diversity and density. Additionally, this anatomical location has other health-related functions, including extensive carbohydrate metabolism [16], which may play a role for the BW phenotype. The cecal contents were analysed by deep shot gun metagenomic sequencing using a combinational approach of short and long read sequencing by employing the Illumina and Oxford Nanopore Technologies (ONT) sequencing platforms. The aim was to provide a real-life-relevant insight into the question of why some broilers grow faster than others. We hypothesized that broilers of different size have different microbiome characteristics, despite being housed in the same pen.

## Results

### Body weight on day 37 spanned from 1514 g to 3134 g

**Fig. 1:**
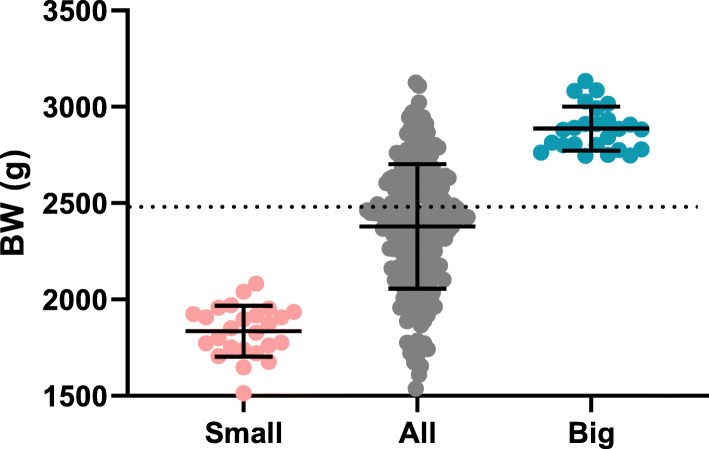
BW after 37 days of rearing for the flock of 211 birds and for the 25 lightest and 25 heaviest birds selected for microbiome analysis. Dotted line indicates expected BW according to breeder’s performance objectives, solid lines indicate mean BW with SD. BW = body weight

At placement in the barn, bird mean BW was 45.5 g. After 37 days, birds averaged 2379 g, which was about 8% below (or 2 days behind) the breeder’s performance objective of 2592 g [17]. The 25 heaviest birds (Big) averaged 2887 g with the biggest bird achieving 3134 g. The 25 lightest birds (Small) had a mean BW of only 1836 g and the smallest one weighing 1514 g (Table 1; Fig. 1). The standard deviation (SD) within the two groups was not statistically significantly different (115 g versus 132 g in the Big and Small birds, respectively; *p* = 0.51; F-test of equal variances). However, if put on a relative scale, BW of Big birds were actually more uniform than Small birds (CV of 4% vs. 7%). Over the 37 days period, losses and culls amounted to 3.2%. Foot pad lesions were scored on day 37, and there was no difference (*p* = 0.14) between Big and Small birds (data not shown).

**Table 1:** Descriptive statistics of body weight after 37 days of rearing. The incongruence between the min-max values for “All” birds (1538–3126 g) versus the Min value for Small birds (1514 g) and Max value for Big birds (3134 g) is explained by a time-difference of up to 6 h in between weighing #1 of all birds leading to the sorting of the 25 heaviest and 25 lightest birds and weighing #2 of the subset of 50 birds immediately before sacrificing

	All	Small	Big
n	211	25	25
Mean (g)	2379	1836	2887
Standard deviation (g)	323	132	115
Standard error of the mean (g)	22	26	23
Coefficient of variation (%)	13.56	7.16	3.98
Min (g)	1538	1514	2745
Max (g)	3126	2082	3134
Relative range (%)	67	31	13

### Combination of short and long read sequencing yielded superior quality of metagenomes

All 50 cecal samples were sequenced by Illumina short read sequencing, while 10 samples from five Big and five Small birds were additionally sequenced by ONT long read sequencing. DNA extraction, library preparation and DNA sequencing using Illumina was successful for all 50 samples and generated 4.8 to 35 Gbp of data after trimming (Supplementary Table S3). To make sure the ONT and Illumina sequencing results were comparable, the same DNA was used for both platforms. The 10 ONT samples were sequenced on five Nanopore MinION flowcells and, after trimming away reads less than 1000 bp, yielded between 4.1 and 15.3 Gbp of data available for metagenome assembly (Supplementary Table S2). After dereplication, 43 high-quality (HQ) and 128 medium-quality (MQ) Metagenome Assembled genomes (MAGs) were obtained. A number of the MAGs even assembled in single contigs, representing complete genomes. The dereplicated MAG dataset captured 26.5 to 84.7% of the ONT data and 17.6 to 84.4% of the Illumina data. Taken together, a high amount of data in the individual MAGs was captured - with most data contained in HQ MAGs. 

### Big broilers had the highest microbial diversity and displayed microbiome uniformity

**Fig. 2:**
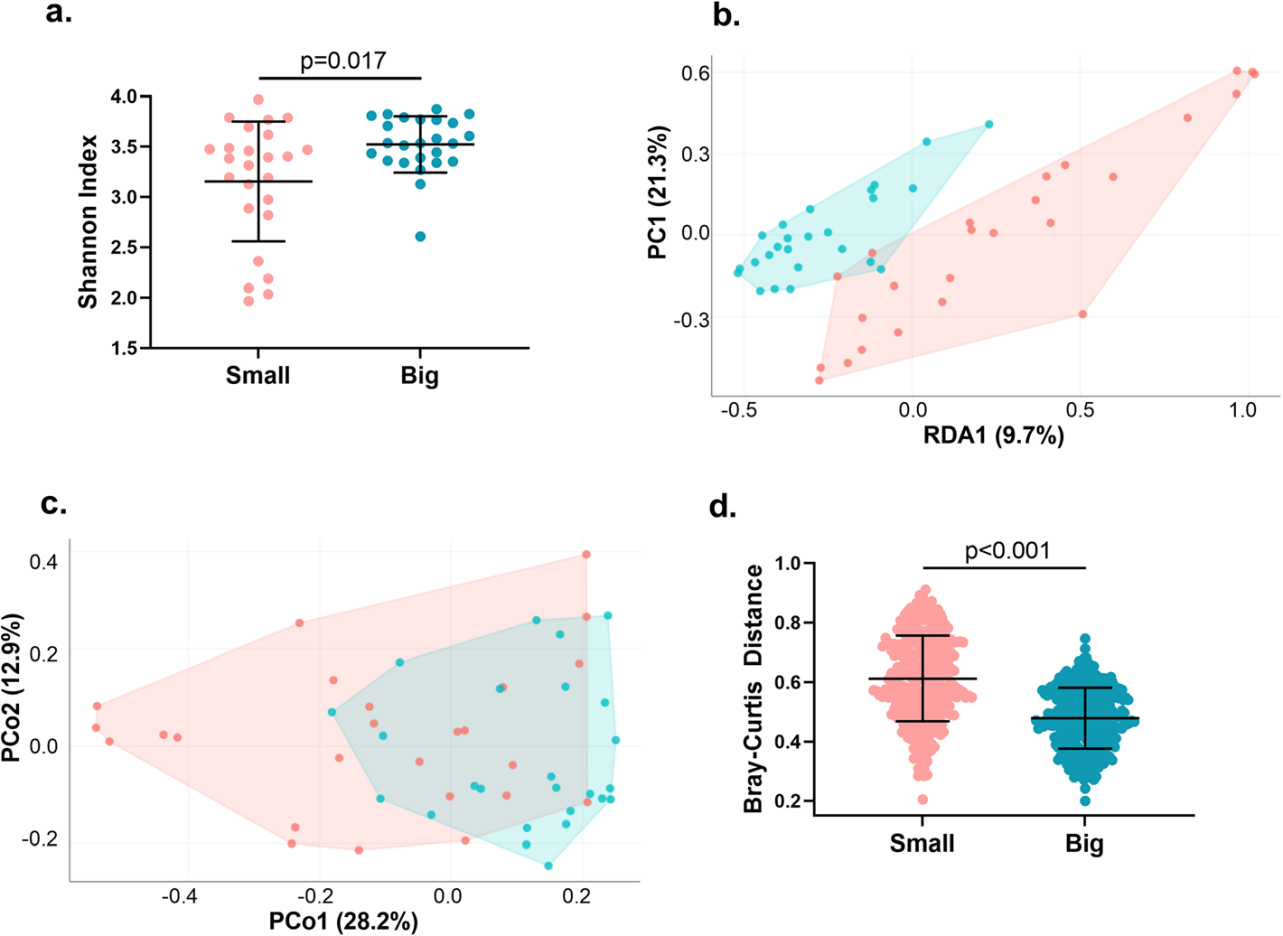
**a** Alpha diversity (Shannon Index) of ceca of Small and Big broilers. Big birds had significantly higher diversity (*p* = 0.017; Wilcoxon rank sum test). b Beta diversity (RDA ordination) plot of cecal samples constrained to the variable “Small/Big”. An almost complete separation between the groups is observed. Less interindividual variation occur between the big birds compared to the small birds. The relative contribution (eigenvalue) of each axis to the total inertia in the data as well as to the constrained space only, respectively, are indicated in percent at the axis titles. **c** Beta diversity **(**PCoA) plot based on Bray-Curtis dissimilarity matrix. The relative contribution (eigenvalue) of each axis to the total inertia in the data is indicated in percent at the axis titles. **d** Bray-Curtis dissimilarity in Small and Big chickens. The interindividual variation was tested using by pair-wise comparisons of within-group Bray-Curtis dissimilarities. The values can be between 0 and 1, with 0 meaning identical communities and 1 meaning there is no overlap in the communities. Significance was tested using non-parametric Wilcoxon rank sum test. RDA = Redundancy Analysis. PCoA = Principal Coordinates Analysis. Solid lines indicate mean with SD

The alpha diversity (diversity within each sample) as measured by the Shannon Index was significantly higher in the Big birds compared to the Small birds (*p* = 0.017; Fig. 2a). Beta diversity (diversity between samples) as assessed by Redundancy Analysis (RDA) constrained to the classification of Big versus Small chickens revealed a strong separation between the two groups (Fig. 2b). Assessment of beta diversity by Principal Coordinates Analysis (PCoA) applying the Bray-Curtis dissimilarity demonstrated a partial separation between the groups (Fig. 2c). For the RDA as well as the PCoA plot, the microbiomes of the Big birds displayed less bird-to-bird variation compared to the Small birds, i.e. the microbiomes were more uniform in the Big birds. Testing by pair-wise comparisons of the Bray-Curtis dissimilarities within the same size group confirmed a significantly smaller variation between samples from the Big chickens (*p* < 0.001; Fig. 2d).

### Taxa across all taxonomic ranks were differentially abundant between small and big broilers

**Fig. 3:**
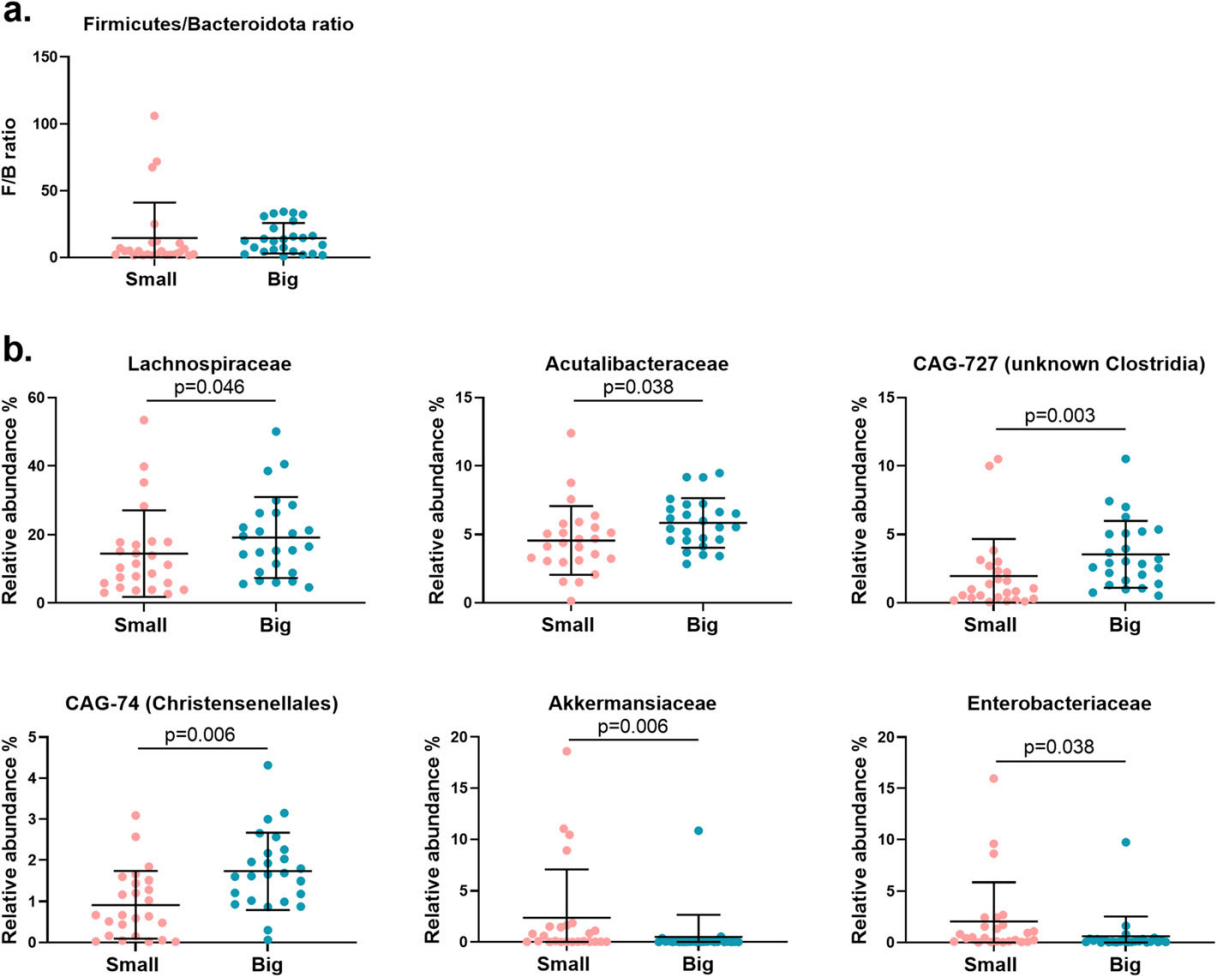
**a** *Firmicutes*/*Bacteroidota* ratio of Big and Small broiler chickens (*p* > 0.05; t-test on log2-transformed data). **b** Differentially abundant families between Small and Big chickens. Differences were tested by calculating the fold change of log2-transformed relative abundances of Big relative to Small. Solid lines indicate mean with SD

**Fig. 4:**
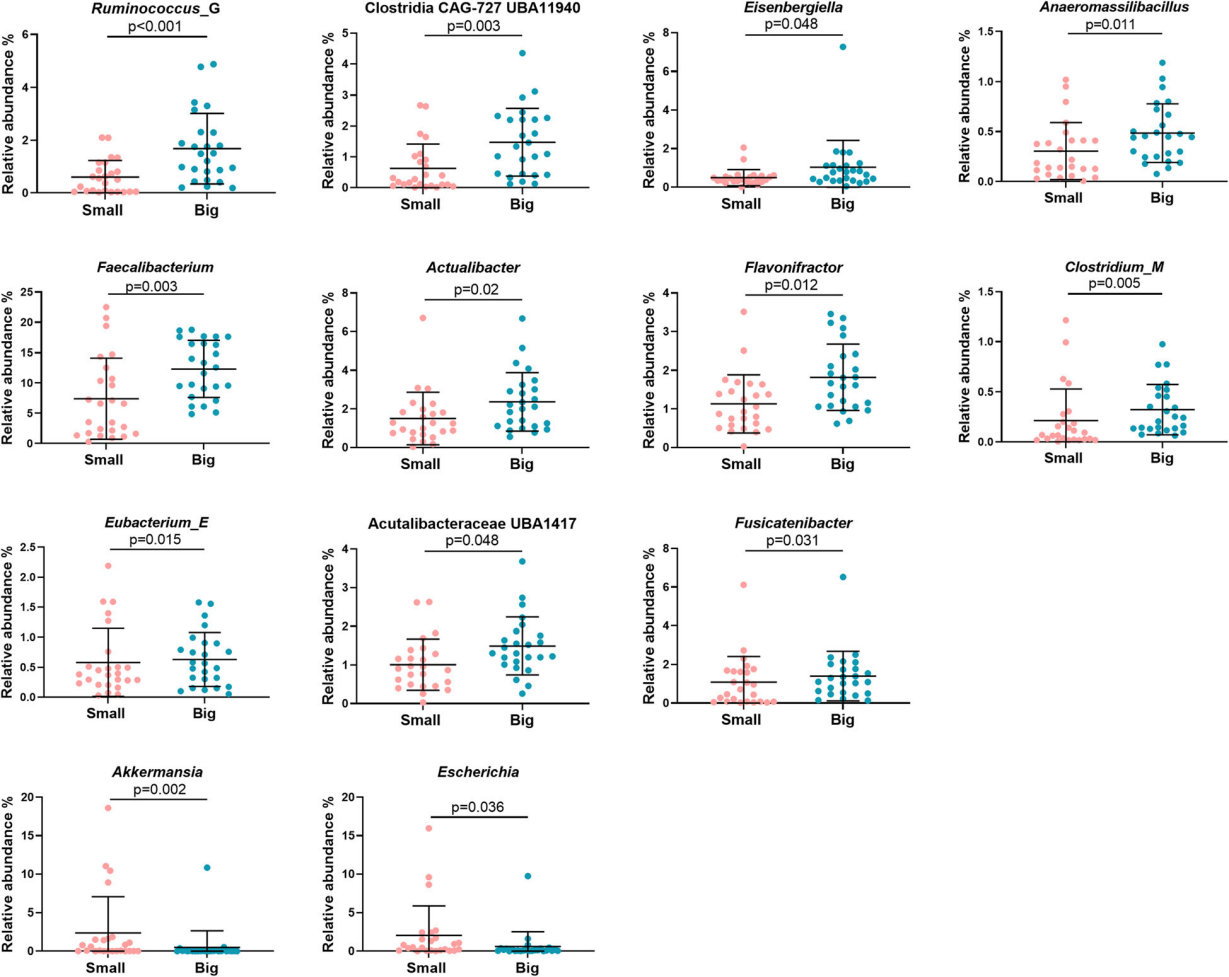
Significantly different genera between Big and Small broiler chickens^1^. Differences were tested by calculating the fold change of log2-transformed relative abundances of Big relative to Small. Solid lines indicate mean with SD. ^1^ not all genera are referred to in the text

Differences between the Big and Small chickens were investigated using a descending taxonomic rank approach, starting from phylum rank for a high level overview, then family, genus and ultimately the MAG level, which is comparable to species level or in some cases even strain level. Testing of the differentially abundant taxa was done by calculating the fold change of log2-transformed relative abundances of Big chickens relative to Small chickens. Six phyla were represented across all samples: *Firmicutes, Actinobacteriota, Bacteroidota* (also known as *Bacteroidetes), Verrucomicrobiota, Proteobacteria* and *Cyanobacteria*. *Firmicutes* was the dominating phylum both in Big birds (66 ± 13% SD) and Small birds (58 ± 15% SD; Supplementary Table S5). Among all phyla, *Verrucomicrobiota* (*p* = 0.004) and *Proteobacteria* (*p* = 0.03) were more abundant in Small birds (not shown), while no phylum was more abundant in the Big birds. The *Firmicutes*:*Bacteroidota* (F/B) ratios were not different between Big and Small (Fig. 3a). Four families were more abundant in Big birds: *Lachnospiraceae* (*p* = 0.046), *Acutalibacteraceae* (*p* = 0.038), an unknown *Clostridia* family classified as CAG-727 (*p* = 0.003) and a family classified as CAG-74 (*p* = 0.006) from the *Christensenellales* order (Fig. 3b). Two families were significantly more abundant in Small birds: *Akkermansiaceae* (phylum *Verrucomicrobiota; p* = 0.006) and *Enterobacteriaceae* (phylum *Proteobacteria*; *p* = 0.038; Fig. 3b). Eleven genera were more abundant in Big birds, for instance *Faecalibacterium* (*p* = 0.003), *Eisenbergiella* (*p* = 0.048), *Flavonifractor* (*p* = 0.012) and *Ruminococcus* (*p* < 0.001; Fig. 4). As was also reflected on the phylum and family level, two genera were more abundant in the Small birds, *Akkermansia* (*p* = 0.008) and *Escherichia* (*p* = 0.038; Fig. 4).

Five MAGs were significantly more abundant in Small birds, while 31 MAGs had a higher abundance in Big birds (Table 2). MAGs enriched in Small birds were one *Gemmiger* MAG (*p* = 0.006), one *Blautia* MAG (*p* = 0.012), one *Ruthenibacterium* MAG (*p* = 0.05), *Akkermansia muciniphila* (*p* = 0.006) and *Escherichia coli* (*p* = 0.036). The two latter MAGs were driving the differences as described for the Small birds on the phylum, family and genus levels (Fig. 3+ 4). Of the 31 MAGs with highest abundance in the Big birds, the majority were from the *Lachnospiraceae* (11 MAGs) and *Ruminococcaceae* (7 MAGs). The remaining 13 MAGs were distributed to two MAGs from the *Christensenellales* order, three MAGs from *Butyricicoccaceae* (two *Butyricicoccus* and one *Agathobaculum*), three *Acutalibacteraceae* MAGs, two *Oscillospiraceae* and one from *Bifidobacteriaceae* (*Bifidobacterium gallinarum*) and two not further classified MAGs from the Clostridia class termed CAG-727 (see *p*-values in Table 2).

**Table 2:** Significantly different MAGs in Big and Small broilers

	MAG	Phylum	Family	Genus	log2FC	Adjusted ***p***-value	Avg Small (%)	Avg Big (%)
**Highest abundance in Small chicken**	HQ/98/3-BC04_bin.5	Firmicutes	*Ruminococcaceae*	*Gemmiger*	−5.19	0.006	6.19	0.17
	HQ/94/0-BC04_bin.119	Firmicutes	*Lachnospiraceae*	*Blautia*	−3.97	0.012	0.47	0.03
	MQ/83/3-BC08_bin.49	Firmicutes	*Ruminococcaceae*	*Ruthenibacterium*	−2	0.05	0.28	0.07
	HQ/98/0-BC08_bin.2	Verrucomicrobiota	*Akkermansiaceae*	*Akkermansia*	−1.86	0.006	0.91	0.25
	HQ/100/0-BC01_bin.62	Firmicutes	*Enterobacteriaceae*	*Escherichia*	−1.76	0.036	0.95	0.28
**Highest abundance in Big chicken**	MQ/85/0-BC06_bin.111	Firmicutes	*Clostridia* CAG-727	UBA11940	3.30616	< 0.001	0.17	0.56
	MQ/83/1-BC03_bin.150	Firmicutes	*Christensenellales* CAG-74	*–*	2.963665	< 0.001	0.12	0.33
	MQ/53/0-BC05_bin.97	Firmicutes	*Butyricicoccaceae*	*Butyricicoccus*	2.522999	0.005	0.07	0.14
	MQ/51/2-BC03_bin.153	Firmicutes	*Butyricicoccaceae*	*Butyricicoccus*	2.487598	0.003	0.1	0.19
	MQ/77/1-BC06_bin.26	Firmicutes	*Lachnospiraceae*	*Blautia*	2.442135	0.005	1.31	2.29
	HQ/93/1-BC09_bin.81	Firmicutes	*Lachnospiraceae*	*Ruminococcus_G*	2.338411	< 0.001	0.15	0.48
	HQ/92/1-BC01_bin.127	Firmicutes	*Lachnospiraceae*	*Ruminococcus_G*	2.267726	< 0.001	0.11	0.33
	MQ/78/2-BC01_bin.64	Firmicutes	*Oscillospiraceae*	*Flavonifractor*	2.137273	< 0.001	0.18	0.42
	MQ/81/1-BC05_bin.63	Firmicutes	*Ruminococcaceae*	*Gemmiger*	2.103554	0.006	0.15	0.42
	MQ/64/0-BC06_bin.117	Firmicutes	*Oscillospiraceae*	*Flavonifractor*	1.997778	< 0.001	0.14	0.39
	MQ/65/2-BC08_bin.109	Firmicutes	*Acutalibacteraceae*	*Acutalibacter*	1.992992	0.003	0.05	0.15
	MQ/82/0-BC01_bin.108	Firmicutes	*Lachnospiraceae*	*Eubacterium_E*	1.793982	0.015	0.13	0.21
	HQ/95/3-BC05_bin.99	Firmicutes	*Ruminococcaceae*	*Faecalibacterium*	1.738848	0.001	0.97	2.01
	HQ/96/0-BC10_bin.135	Firmicutes	*Clostridia* CAG-727	*–*	1.69629	0.03	0.47	0.89
	MQ/65/0-BC06_bin.35	Firmicutes	*Lachnospiraceae*	*Eisenbergiella*	1.693181	0.042	0.07	0.24
	MQ/58/1-BC03_bin.187	Firmicutes	*Lachnospiraceae*	*Clostridium_M*	1.670223	0.005	0.09	0.15
	HQ/99/0-BC06_bin.137	Firmicutes	*Ruminococcaceae*	*Gemmiger*	1.658808	0.002	0.59	1.99
	MQ/75/1-BC10_bin.88	Firmicutes	*Christensenellales* CAG-74	*–*	1.613336	0.016	0.18	0.38
	MQ/56/0-BC03_bin.169	Firmicutes	*Lachnospiraceae*	*Faecalicatena*	1.603117	0.026	0.1	0.15
	MQ/67/1-BC09_bin.14	Firmicutes	*Lachnospiraceae*	*Fusicatenibacter*	1.580042	0.036	0.08	0.14
	HQ/92/1-BC08_bin.120	Firmicutes	Unknown *Lachnospiraceae*	*–*	1.568899	0.042	0.18	0.34
	HQ/97/1-BC06_bin.90	Firmicutes	*Ruminococcaceae*	*Faecalibacterium*	1.476226	0.002	1.1	2.12
	MQ/66/0-BC08_bin.61	Firmicutes	*Ruminococcaceae*	*Gemmiger*	1.457215	0.015	0.08	0.19
	MQ/89/1-BC03_bin.47	Firmicutes	*Lachnospiraceae*	*–*	1.399621	0.044	0.2	0.32
	HQ/96/2-BC04_bin.170	Firmicutes	*Lachnospiraceae*	*Fusicatenibacter*	1.309454	0.036	0.3	0.42
	MQ/68/1-BC02_bin.193	Firmicutes	*Ruminococcaceae*	*Faecalibacterium*	1.307194	0.013	1.26	1.91
	MQ/84/2-BC06_bin.56	Firmicutes	*Butyricicoccaceae*	*Agathobaculum*	1.278165	0.037	0.17	0.23
	MQ/80/0-BC08_bin.25	Firmicutes	*Ruminococcaceae*	*Gemmiger*	1.25775	0.034	0.64	1.35
	HQ/99/0-BC06_bin.15	Actinobacteriota	*Bifidobacteriaceae*	*Bifidobacterium*	1.118436	0.044	2.79	4.76
	HQ/97/0-BC05_bin.93	Firmicutes	*Acutalibacteraceae*	UBA1417	1.030908	0.029	0.12	0.22
	MQ/68/0-BC06_bin.99	Firmicutes	*Acutalibacteraceae*	UBA1417	0.968859	0.05	0.09	0.17

*MAG* Metagenome Assembled Genome. *MQ* Medium quality genome, *HQ* High quality genome

## Discussion

In this study, a hybrid metagenomic sequencing approach demonstrated a likely prominent role of the cecal microbiota in broiler growth and body weight heterogeneity.

### Alpha diversity

The Big birds had a higher alpha diversity compared to the Small birds from the same pen. Microbial diversity of the gastrointestinal tract is a solid marker of gut health, as mostly demonstrated in humans [18]. In contrast, lower diversity is a marker of dysbiosis and is a consistent finding in patients suffering from inflammatory and immunological conditions [18]. In poultry, high diversity has also been linked to high productivity and gut health [8]. Increased microbial diversity translates to diversity of the microbial gene pool, which ultimately should result in presence of different types of organisms providing beneficial pathways to the host. The high alpha diversity and thus improved microbiome functionality could therefore have contributed to the improved growth of the Big chickens in our study.

### Microbiome uniformity

We found that the Big birds had more uniform microbiome compositions within the group of birds than the Small birds displaying much more variable microbiomes. Microbiome variability has previously been linked to disease in various ecological systems leading researchers to coin a so-called Anna Karenina principle (AKP) for animal microbiomes [19, 20]. The AKP says that “*all healthy microbiomes are alike; each dysbiotic microbiome is dysbiotic in its own way*”, which is referring to the opening line from Leo Tolstoy’s novel Anna Karenina: “*All happy families are alike, each unhappy family is unhappy in its own way*”. It means that the more similar (high uniformity) individuals of a population are microbiome-wise, the lower is the risk of dysbiosis and disease within the population [20]. We found a significantly higher microbiome uniformity (lower beta-diversity) between the Big birds in our study despite the shared environment with the Small birds. This supports the hypothesis that healthy microbiomes (which in turn can lead to better growth) are more similar than less healthy or even dysbiotic microbiomes (which in turn can inhibit optimal growth). This suggests that AKP effects can develop within sub-populations of a larger population. The higher microbiome uniformity of the Big birds in our study was not statistically mirrored by a higher BW uniformity, though the BW CV as well as the SD were numerically lower in the Big birds. These data support further investigation regarding the relationship between microbiome uniformity and phenotypic uniformity in sufficiently powered trials in terms of sample size and replicates allowing for measurements of feed intake, daily BW gain and feed conversion ratio calculations.

### Bacterial taxa related to health and productivity

Many taxa from *Firmicutes* were more abundant in the Big chickens, but the F/B ratio was not different between Big and Small broilers. A high F/B ratio has been associated with improved energy harvest and thus improved productivity in production animals [21–23]. Alternatives to antibiotic growth promoters such as probiotics and plant extracts can increase the F/B ratio and are shown to correlate positively with BW in broilers [22, 23]. It has, however, been reported that the F/B ratio varies substantially between individuals [24, 25] and the reliability of the F/B ratio as an productivity marker has been questioned [25, 26].

Several SCFA-producing taxa were dominant in the Big chickens compared to Small. Among these were bacteria from *Lachnospiraceae*. The metabolite profiles of *Lachnospiraceae* varies between species, but all of them produce SCFAs, expectedly being beneficial to the host [9, 27]. The SCFA butyrate is specifically a critical energy-source for the colonocytes and exerts numerous beneficial effects, including growth promotion, antimicrobial activity, immunomodulation and anti-inflammatory activity; inclusively these attributes can lead to a reduction in pathogens [28]. Of butyrate-producing taxa with higher levels in the Big birds were *Eisenbergiella* [29], *Eubacterium*, *Faecalicatena* [30] and *Flavonifractor* [31]. *Flavinofractor* could in addition possess regulatory immunomodulatory properties [32]. *Agathobaculum*, a more recently isolated taxon with promising effects due to butyrate-production [33] were also enriched in the Big birds. Less is known about the *Acutalibacteraceae* MAGs which were also more abundant in the Big chickens. It is a novel and relatively undescribed taxon [34] and to our knowledge has not previously been identified in poultry before. MAGs of the *Gemmiger* genus were also more abundant in the Big chickens, while also one *Gemmiger* MAG was more abundant in the Small. *Gemmiger* species are common inhabitants of the avian intestinal tract, where they produce an array of acids including formate and butyrate [35, 36]. *Faecalibacterium* was more abundant in the Big chickens, which is in alignment with the findings of Lee et al. who also reported an enrichment of this genus in high BW male chickens [11]. *Faecalibacterium prausnitzii*, the only known species within the genus, is a highly effective butyrate-producer [37] and several other reports link these probiotic organisms to high chicken productivity [8]. On the other hand, *Ruthenibacterium* were more abundant in the Small birds and was recently described as primarily lactate-producers with butyrate as the other major SCFA end product [38]. There was an increased presence of *Butyricicoccus* in the Big birds. This taxon contains well-known butyrate-producers with strong anti-inflammatory effects in several hosts including broilers [39]*.*

Among other SCFA-producing taxa enriched in the Big chickens were *Fusicatenibacter* which produce formate and acetate [40]. Counterintuitively, two *Blautia* MAGs were more abundant in the Small and Big chickens, respectively. *Blautia* metabolize undigested carbohydrates resulting in the production of acetate [27, 41]. Microbial-derived acetate may be involved in regulating BW and satiety [42]. *Blautia* were more abundant in lean compared to fat broiler lines [43] and is considered a keystone health-associated anti-inflammatory taxon of the human microbiome [27]. There could be species- or strain-level relevant differences causing different effects in the host, which could explain the association of *Blautia* with both the Big and the Small chickens. Importantly, it must also be remarked that an association between gut bacteria and host traits cannot be used to conclude causal relationships.

Finally, we found that the family designated as CAG-74 from the *Christensenellales* order and *Bifidobacterium gallinarum* had a higher abundance in the Big chickens compared to Small. *Christensenellales* is related to *Christensenellaceae,* a family strongly associated with health in humans [44]. *Christensenellaceae* has also been reported to increase during probiotic supplementation in broilers [45] and was associated with high feed efficiency in a study with pigs [46]. Bifidobacteria are well-known for their beneficial effects for the host [47], yet the specific role of *B. gallinarum* in broiler productivity is to our knowledge unknown.

*E. coli* and *A. muciniphila* were both more abundant in the Small birds. *E. coli* is involved in localized and systemic infections in production poultry termed colibacillosis, which is worldwide a major cause of economic loss and compromising of animal welfare and food safety [48].. A*. muciniphila* is a mucin-degrader and a robust, positively correlated marker of metabolic health and leanness in humans [49]. *Akkermansia* has previously been negatively correlated with BW in broilers [12]. Interestingly, the genus has also been demonstrated to be in higher abundance in chickens with good feed efficiency (FE) compared to chickens with poorer FE [50]. *A. muciniphila* has a regulatory role in lipid metabolism [51], providing one possible explanation why birds with highest *A. muciniphila* abundance in our study had the lowest BW. We did not investigate fat content of the carcasses, but it could had been a relevant parameter to include.

Everything taken together, there was unarguably an increased presence of taxa related to health and performance in the Big chickens, which may have contributed to the better growth due to anti-inflammatory and feed utilization properties. As a further investigation for establishing a causal relationship between the microbiome composition and BW phenotype, one would need to transplant the phenotype-associated microbiomes into germ-free chicks and monitor the BW development.

### What causes microbiome heterogeneity within a population with shared environment?

It is well-known that age, sex, husbandry and management practices, season, circadian rhythm, geography etc. can cause microbiome variation between populations [13, 14]. Due to our one-pen study design mimicking a production broiler house on a smaller scale, the enormous microbiome differences we observed between Big and Small broilers cannot be attributed to such confounding effects. In a production broiler flock, with housing of hundreds of square meters, it is feasible that local shared environments could develop among birds as they primarily segregate in one section of the house. The birds in this study, on the other hand, were housed in a relatively small pen (13.5 m^2^) and due to coprophagy and pecking/preening behaviour, we assume that there was a relatively equal microbe sharing across the pen.

Foot pad lesion scores were not different between Big and Small, hence this factor could be ruled out as a major cause for growth differences between birds. Social stress and a reduction of feed intake by lower ranking birds can be relevant to consider. We accounted for this by providing sufficient feeder space, approximately twice the amount of commercial standards (i.e. 2.98 cm per bird or 1.25 cm/kg BW). However, with our study design, individual feed intake was not measured. Thus, we cannot rule out that stress and hierarchy factors were contributing to variable feed intake resulting in growth heterogeneity.

The study aimed at providing the same conditions to each of the birds from placement to slaughter, but differences in intestinal microbiomes may have been seeded prior to placement in the barn. Contributions could have been made by hen (in ovary seeding), hen environment and egg handling (trans-shell seeding), incubation and hatching environment (trans-shell seeding and initial oral intake) as well as transport of chickens to the barn (oral intake). The influence of hatcher was minimized in this trial as all chicks came from the same hatcher. Nevertheless, despite disinfection practices at the hatchery, some bacteria could still have crossed the egg shell [52] during lay and handling before disinfection, which could have shaped the microbiome of the hatched chicks [53].

Genetics cannot be ignored as a factor in shaping the microbiome, though the genetic effect expectedly is smaller than the environmental [4, 5]. Residual heterozygosity and polymorphisms within the broiler line can contribute to microbiome variation and phenotypic variation [54, 55].

Ultimately, the largest influencer on microbiome heterogeneity in individuals within a shared environment can probably be ascribed to stochastic events, i.e. the randomness in which microbes each chick encounters first in its life. A self-enforcing cascade of events might happen: some birds have by chance poorer microbiome, leading to reduced nutrient absorption and the birds not feeling as fit as the others. This can lead to social stress by lower ranking and a viscous circle of reduced feed intake and picking in the litter with increased ingestion of pathogenic and dysbiosis-related bacteria starts. Ultimately, this leads to an even more suboptimal microbiome. At some point, the difference in BW itself will force further differentiation, as larger birds are requiring more feeder space for a longer duration of time. These heavy broilers will be harder to push aside when lighter birds want to make their way to the feeder and drinker.

Beneficial early life programming of the gut microbiome is crucial for the development of the immune system, establishment of gut health and even behavior [56, 57]. Such programming may be achieved through probiotic delivery methods such as in ovo, trans-shell seeding, feed additives or seeding of the chick environment. These intervention examples might also be helpful in overcoming microbiome heterogeneity and thus BW heterogeneity.

## Conclusions

We present here a controlled study in which the gut microbiome composition of male broilers of varying BW housed and reared under the same conditions was investigated. Birds with highest BWs displayed microbiome features coinciding with health benefits: higher microbial diversity, more microbiome uniformity, higher levels of SCFA-producing bacteria and lower levels of the leanness-associated and pathogenic bacteria compared to birds with the lowest BWs.

Our data indicates a strong association between broiler flock heterogeneity and the cecal microbiome composition. Investigations of methods for establishing uniform and beneficial microbiomes of chicks in early life with the aim to improve flock uniformity are therefore warranted.

## Methods

### Broiler management

Two hundred eighteen male, newly hatched Ross 308 broiler chickens were obtained from a commercial hatchery (Geflügelhof Möckern, Germany). At the hatchery, eggs were disinfected upon arrival in the hatchery, immediately prior to placement in the incubator and again at placement in the hatcher unit on day 18. After hatch, the chicks received 1/2 a dose of Infectious bronchitis vaccine (spray-on). Upon arrival in the test facility (feedtest, Wettin-Löbejün Germany), chicks were placed in a 13.5 m^2^ floor pen with mesh panels in a cleaned and disinfected barn, which had been empty for 4 weeks prior to study start. Softwood shavings (HVT Premiumspan Profi, Hobelspanverarbeitung GmbH, Dittersdorf, Germany) served as bedding material. Water was supplied in four bell drinkers. Feed was initially offered in four round hanging feeders (40 cm diameter), after 4 weeks the number of feeders was increased to five. The feed consisted mainly of wheat, soybean meal, rapeseed meal, corn and rye (from week 3), and was supplemented with oil, minerals and free amino acids. Throughout the study period, the feed was supplemented with a coccidiostat (Monteban G100 (active ingredient: narasin), by Elanco at 0.06%) as well as a commercial phytase (HiPhos by DSM at 500 FYT/kg). The ingredients, proportions and the feed additives were included to mimic industry conditions. Feed and water were available ad libitum. Temperature and light were managed according to breeder’s recommendations along with animal welfare requirements. At the age of 15 days, birds were routinely vaccinated against Newcastle disease (Hipraviar) and Infectious bursal disease (Hipragumboro) via drinking water. After 1 week, three birds had to be culled due to not growing (BW < 100 g) and obvious signs of poor health. A fourth bird was culled due to leg deformation at 32 days of age. Another three birds died (no necropsy conducted) within the initial 2 weeks of the study. Otherwise, there were no veterinary interventions throughout the study. Neither feed consumption nor BW during the rearing period were recorded.

### Cecal sampling

At the age of 37 days, all birds were weighed individually and subsequently ranked in ascending order (=weighing #1). The 25 heaviest and the 25 lightest birds were selected for sampling. These 50 individuals were temporarily put into smaller pens with clean bedding material and continued on the original diet until sampling to ensure sufficient intestinal filling. Groups of four birds were then forwarded to sampling. Time from placement in the smaller pens until sacrificing was 1–6 h. After weighing again (=weighing #2) and assignment of sample ID, birds were stunned and exsanguinated. After opening the abdominal cavity, one cecal sac per bird was sampled. The sacs were closed by staples to prevent leakage and transferred into labelled containers and placed in a − 80 °C freezer. Average lag time between slaughter and freezing of the samples was 14 min. Sample containers were shipped to the analyzing laboratory on dry ice and placed in a − 80 °C freezer until analysis.

### Microbiome analyses

DNA extraction, library preparation, sequencing and bioinformatics/statistics on metagenomic data was performed by DNASense Aps (Aalborg, Denmark). To obtain high quality metagenomic data, shot gun short read deep sequencing (Illumina) was applied on all 50 cecal samples. Ten samples from five Small and five Big birds were additionally sequenced by long read sequencing using Oxford Nanopore Technology (ONT) and deep Illumina sequences used for error-correction. Each of the samples were assembled, binned and polished individually and dereplicated to a non-redundant set of reference Metagenome Assembled Genomes (MAGs). These dereplicated MAGs were used as a reference database and Illumina and ONT data mapped to measure abundance across all 50 samples.

### DNA extraction

The stapled cecal sacs were thawed, emptied and the digesta homogenized in sodium phosphate buffer. DNA extraction was performed using the standard protocol for FastDNA Spin kit for Soil (MP Biomedicals, USA) with the following exceptions: 500 μL of sample, 80 μL sodium phosphate buffer and 120 μL MT Buffer were added to a Lysing Matrix E tube. Bead beating was performed at 6 m/s for 4x40s. Gel electrophoresis using Tapestation 2200 and Genomic DNA screentapes (Agilent, USA) were used to validate product size and purity of a subset of DNA extracts. DNA concentration was measured using Qubit dsDNA HS/BR Assay kit (Thermo Fisher Scientific, USA).

### Illumina metagenomic library preparation and sequencing

Sequencing libraries were prepared using the NEB Next Ultra II DNA library prep kit for Illumina (New England Biolabs, USA) following the manufacturer’s protocol. The sequencing libraries were pooled in equimolar concentrations and diluted to 4 nM. The samples were paired end sequenced (2x151bp) on a HiSeq (Illumina, USA) following the standard guidelines for preparing and loading samples on the HiSeq.

### ONT metagenomic library preparation and sequencing

Sequencing libraries were prepared using the LSK109 protocol with native barcoding (Oxford Nanopore Technologies, UK) following the manufacturer’s protocol. The sequencing libraries were sequenced on R9.4.1 flow cells (Oxford Nanopore Technologies, UK) following the manufacturer’s protocol. The reads were base called using Guppy with high accuracy mode.

### Metagenome assembly, quality of genomes and binning

The Illumina sequence reads were trimmed for adaptors using cutadapt (v. 1.16, [58]). The ONT reads were trimmed for adaptors using Porechop (v. 0.2.4, [59]). Reads below 1000 bp were removed. The nanopore reads were assembled independently for each sample using flye (v. 2.6, [60]). The assembly was polished with ONT reads using minimap2 (v. 2.12-r827 [61]), and racon (v. 1.3.3 [62]), followed by polishing with medaka (v.0.8.1, github.com/nanoporetech/medaka). Then, finally the assembly was polished with Illumina data using minimap2 (v. 2.12-r827 [61]), and racon (v. 1.3.3, [62]). The reads were mapped back to the assembly using minimap2 (v. 2.12-r827 [61]) to generate coverage files for metagenomic binning. Genome binning was carried out using metabat2 (v. 2.12.1, [63]). Genome bins were dereplicated using dRep (v. 2.3.2 [64]). Completeness for the dereplicated bins was estimated using CheckM [65]. Genome bins were classified using the Genome Taxonomy Database (GTDB; v. 0.3.2, [66]). Genome statistics were calculated using QUAST (v. 4.6.3, [67]). rRNA sequences were extracted using barrnapp (v. 0.9, github.com/tseemann/barrnap).

All 50 cecal samples were sequenced by Illumina short read sequencing, while 10 samples from five Big and five Small birds were additionally sequenced by ONT long read sequencing. DNA extraction, library preparation and DNA sequencing using Illumina was successful for all 50 samples and generated 4.8 to 35 Gbp of data after trimming (Supplementary Table S1). A negative control DNA extraction showed very little DNA and very few reads indicating that kit contamination was not an issue. To make sure the ONT and Illumina sequencing results were comparable, the same DNA was used for both platforms. The 10 ONT samples were sequenced on five Nanopore MinION flowcells and yielded an average of 19.58 Gbp raw data, which is on par with the current state-of-art yields per flowcell. However, due to no size-selection and an Illumina optimized DNA extraction a large amount of data was removed in the filtering process, especially for the samples with the lowest read N50 (2502–5556 bp) (Supplementary Figure S1). N50 is the shortest contig length that is needed to cover 50% of the whole genome sequence. Hence, after trimming away reads less than 1000 bp, between 4.1 and 15.3 Gbp of data was available for metagenome assembly (Supplementary Table S2). The individual assemblies were highly dependent on the amount of data after sequencing, but in general all assemblies were good with an order-of-magnitude larger N50 compared to traditional Illumina assemblies (Supplementary Table S3). The percentage of trimmed data mapped to each MAG category for all 60 samples. Quality of the genomes were defined as: HQ = High-Quality genome bin (> 90% complete & < 5% contaminated); MQ = Medium-Quality genome bin (> 50% complete & < 10% contaminated); LQ = Low-Quality genome bin (< 50% complete or > 10% contaminated). After dereplication, 43 high-quality (HQ) and 128 medium-quality (MQ) Metagenome Assembled genomes (MAGs) were obtained. A number of the MAGs even assembled in single contigs, representing complete genomes (Supplementary Table S4). To obtain taxonomy and compare how similar the MAGs were to available reference genomes we used the GTDB workflow to obtain average nucleotide identity (ANI) to the closest reference genome. Most genomes were relatively novel compared to the databases underlining the large benefits of generating genomes directly from the samples. The dereplicated MAG dataset captured 26.5 to 84.7% of the ONT data (avg. 72.2%, Supplementary Table S2) and 17.6 to 84.4% of the Illumina data (avg. 69.1%, Supplementary Table S1). Taken together, a high amount of data in the individual MAGs was captured - with most data contained in HQ MAGs (Supplementary Figure S2).

### Statistical analysis

Metagenomic data were analyzed and visualized through Rstudio [68] using the ampvis2 package [69]. For differential abundance analysis the limma package was used [70]. For redundancy analysis (RDA) the vegan package was used [71]. Prior to downstream analyses, 618 low-quality (LQ) MAGs were removed from the dataset, resulting in 171 medium- and high-quality MAGs. The LQ-MAGs were removed to reduce noise and avoid artefacts introduced by the binning procedure. No apparent outlier samples were detected.

For calculation of Shannon index diversity, MAGs < 0.001 relative abundance were purged from the data set to remove noise from ultra-low abundant MAGs and ensure fair comparison. Significance testing was done by using a non-parametric Wilcoxon rank sum test.

Significance level throughout all analyses was set to 0.05. Prior to RDA and PCoA, MAGs that were not present in more than 0.1% relative abundance in any sample were removed. RDA data were transformed by applying the Hellinger transformation [72]. PCoA was based on Bray-Curtis dissimilarity matrix [73]. Bray-Curtis values used for significance testing by non-parametric Wilcoxon rank sum test were calculated from all pair-wise comparisons of relative abundancies within the same size group. For calculating fold change of MAGs, relative abundances were log2-transformed. Only significant MAGs > 0.1% in average abundance is reported. F/B ratio significance testing was done by log2-transformation and t-test in GraphPad Prism v. 8.4.2 (San Diego, CA, USA). BW of whole flock and sub-populations were analyzed only for descriptive reasons (mean, standard deviation (SD), coefficient of variation (CV), minimum, maximum, relative range). Dispersion of data from the mean is reported as SD throughout the manuscript.

## Supplementary Information

**Additional file 1. Supplementary Figure S1.** Nanopore read length distributions.

**Additional file 2. Supplementary Figure S2.** Percent data recovered in different MAG categories.

**Additional file 3. Supplementary Table S1.** Illumina sequencing statistics.

**Additional file 4. Supplementary Table S2.** Nanopore sequencing statistics.

**Additional file 5. Supplementary Table S3.** Metagenome assembly statistics.

**Additional file 6. Supplementary Table S4.** Genome bin statistics for all High-Quality MAGs.

**Additional file 7. Supplementary Table S5.** Phylum Relative Abundance.

**Additional file 8. Supplementary Table S6.** Family Relative Abundance.

**Additional file 9. Supplementary Table S7.** Genus Relative Abundance.

**Additional file 10. Supplementary Table S8.** MAG Relative Abundance.

**Additional file 11. Supplementary Table S9.** Metadata ENA deposit.

## Data Availability

Metagenomic data generated and analyzed during the current study is deposited in the European Nucleotide Archive with deposit number PRJEB41222. Metadata for linking samples with runID is placed in Supplementary Table S9.

## Abbreviations

AKP: Anna Karenina Principle
BW: Body Weight
CV: Coefficient of variation
FE: Feed Efficiency
HQ: High Quality
LQ: Low Quality
MAG: Metagenome Assembled Genomes
MQ: Medium Quality
PCoA: Principal Coordinates Analysis
RDA: Redundancy Analysis
SCFA: Short chain fatty acid
SD: Standard deviation
