# Supplementary material for: The link between broiler flock heterogeneity and cecal microbiome composition
Source: Anim Microbiome. 2021 Jul 31;3:54. doi: 10.1186/s42523-021-00110-7 (PMC8325257; doi:10.1186/s42523-021-00110-7)
Supplement: Supplementary file 1 — Additional file 1. Supplementary Figure S1. Nanopore read length distributions. [file 42523_2021_110_MOESM1_ESM.docx]

**Supplementary Figure S1.** Nanopore read length distributions

Weighted read length distributions for the 10 samples sequenced with Nanopore. The N50 read length is shown for each sample.


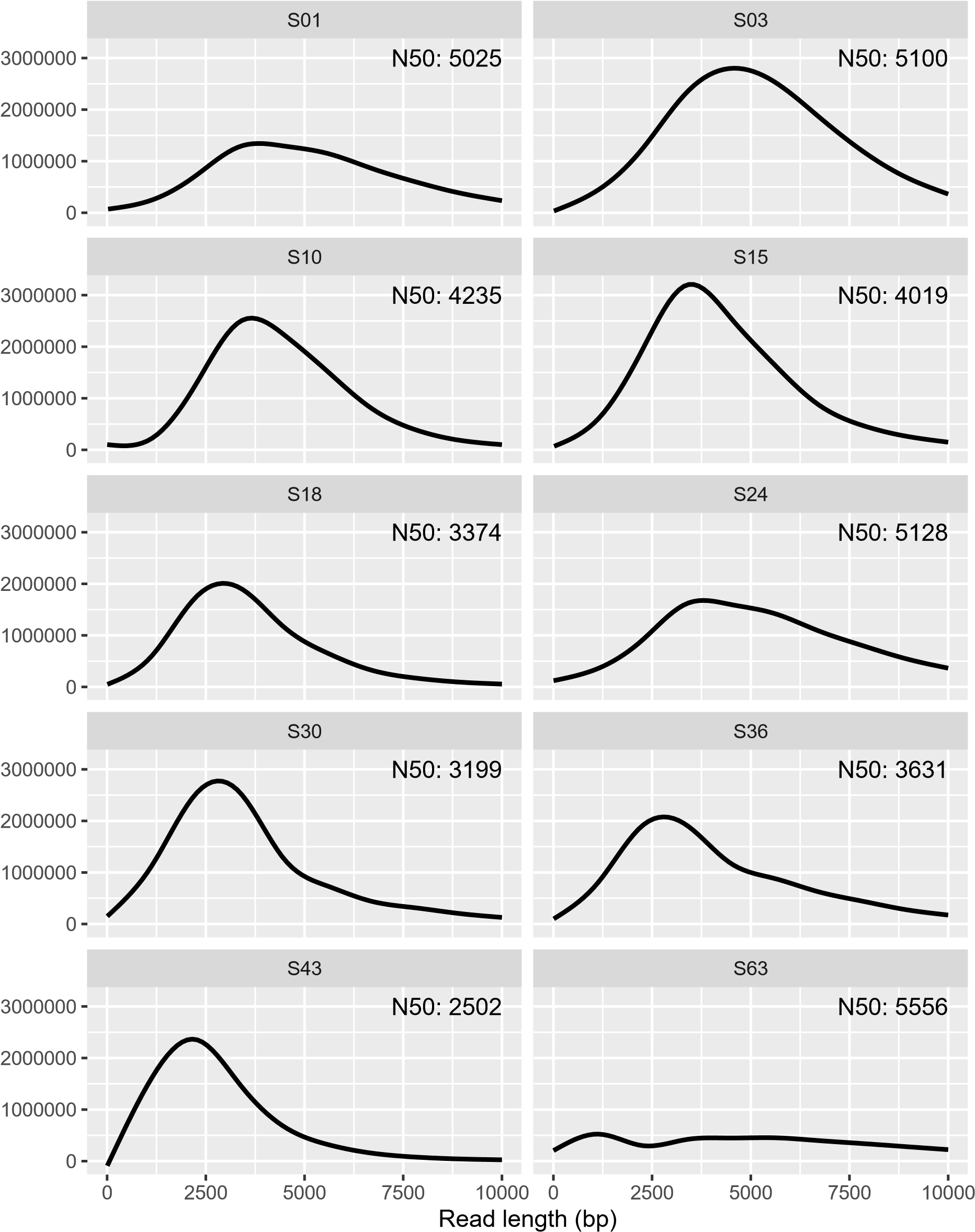


Data volume (bp)
