# Supplementary material for: The link between broiler flock heterogeneity and cecal microbiome composition
Source: Anim Microbiome. 2021 Jul 31;3:54. doi: 10.1186/s42523-021-00110-7 (PMC8325257; doi:10.1186/s42523-021-00110-7)

## Supplementary Figure S2: Percent data recovered in different MAG categories

The percentage of trimmed data mapped to each MAG category for all 60 samples. **_HQ_** = High-Quality genome bin (>90% complete & < 5% contaminated); **_MQ_** = Medium-Quality genome bin (>50% complete & < 10% contaminated); **_LQ_** = Low-Quality genome bin (<50% complete or > 10% contaminated). Note that 10 samples were sequenced with both ONT (Oxford Nanopore Technologies) and ILM (Illumina).


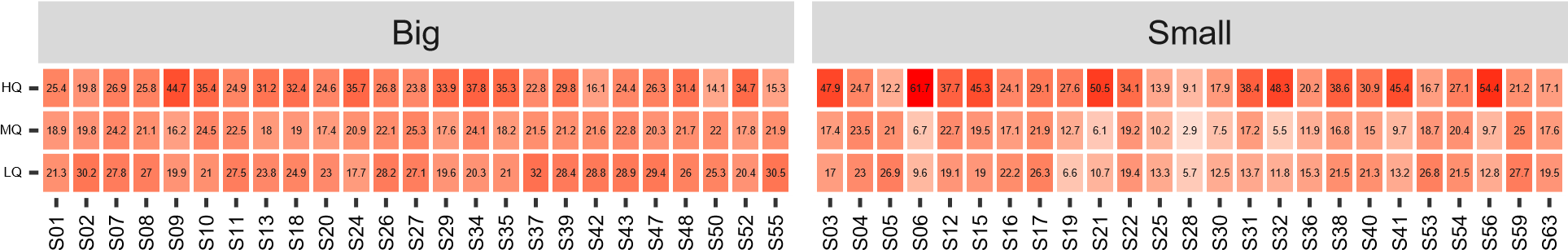

Supplement: Supplementary file 2 — Additional file 2. Supplementary Figure S2. Percent data recovered in different MAG categories. [file 42523_2021_110_MOESM2_ESM.docx]
